# The Transmembrane Domain of CEACAM1-4S Is a Determinant of Anchorage Independent Growth and Tumorigenicity

**DOI:** 10.1371/journal.pone.0029606

**Published:** 2012-01-03

**Authors:** Erica L. Lawson, David R. Mills, Kate E. Brilliant, Douglas C. Hixson

**Affiliations:** Division of Hematology and Oncology, Department of Medicine, Rhode Island Hospital/The Warren Alpert Medical School of Brown University, Providence, Rhode Island, United States of America; Consejo Superior de Investigaciones Cientificas, Spain

## Abstract

CEACAM1 is a multifunctional Ig-like cell adhesion molecule expressed by epithelial cells in many organs. CEACAM1-4L and CEACAM1-4S, two isoforms produced by differential splicing, are predominant in rat liver. Previous work has shown that downregulation of both isoforms occurs in rat hepatocellular carcinomas. Here, we have isolated an anchorage dependent clone, designated 253T-NT that does not express detectable levels of CEACAM1. Stable transfection of 253-NT cells with a wild type CEACAM1-4S expression vector induced an anchorage independent growth *in vitro* and a tumorigenic phenotype *in vivo*. These phenotypes were used as quantifiable end points to examine the functionality of the CEACAM1-4S transmembrane domain. Examination of the CEACAM1 transmembrane domain showed N-terminal GXXXG dimerization sequences and C-terminal tyrosine residues shown in related studies to stabilize transmembrane domain helix-helix interactions. To examine the effects of transmembrane domain mutations, 253-NT cells were transfected with transmembrane domain mutants carrying glycine to leucine or tyrosine to valine substitutions. Results showed that mutation of transmembrane tyrosine residues greatly enhanced growth *in vitro* and *in vivo*. Mutation of transmembrane dimerization motifs, in contrast, significantly reduced anchorage independent growth and tumorigenicity. 253-NT cells expressing CEACAM1-4S with both glycine to leucine and tyrosine to valine mutations displayed the growth-enhanced phenotype of tyrosine mutants. The dramatic effect of transmembrane domain mutations constitutes strong evidence that the transmembrane domain is an important determinant of CEACAM1-4S functionality and most likely by other proteins with transmembrane domains containing dimerization sequences and/or C-terminal tyrosine residues.

## Introduction

CEACAM1 is a member of the carcinoembryonic antigen (CEA) gene family of Ig-like cell-cell adhesion molecules [1], [2]. Like other members of this family, CEACAM1 is a type I transmembrane protein with a heavily glycosylated extracellular region composed of four Ig-like domains, a transmembrane domain and a cytoplasmic tail [3]. In the rat liver there are two allelic variants of CEACAM1 which differ by 16 amino acids in their N-terminal domains [2], [4] and two major splice variants, designated 4L and 4S, that are distinguished by differences in the length of their cytoplasmic tails of 70–72 amino acids and 10–12 amino acids, respectively [2], [4], [5].

Both isoforms of CEACAM1 are down-regulated in epithelial cancers arising in the liver, prostate, bladder and colon [6], [7], [8], [9], [10], [11], [12], a finding that prompted re-expression analysis aimed at defining structure and function relationships. Restoration of expression by infecting rat cell lines derived from primary hepatocellular carcinomas (r-HCC) with a CEACAM1-4L retrovirus resulted in potent growth suppression *in vitro* and tumor suppression *in vivo* [13]. Further analysis showed that the 4L cytoplasmic domain was necessary and sufficient for tumor suppression [14], an activity that required phosphorylation of serine 503 and in colon carcinoma cells, concurrent phosphorylation of tyrosine 488 [15], [16].

In contrast to CEACAM1-4L, CEACAM1-4S failed to generate a tumor suppressor phenotype when re-expressed in r-HCC or mouse colon carcinoma cell lines [13], [17], [18]. However when expressed in MCF7 mouse mammary carcinoma cells, CEACAM1-4S induced glandular morphogenesis, an activity requiring phosphorylation at one or more sites in the 4S cytoplasmic domain [19], [20], [21]. Site directed mutagenesis further showed that mutation of phenylalanine 445 at the C-terminus of the CEACAM1-4S cytoplasmic domain not only compromised interactions with the actin cytoskeleton but also inhibited lumen formation, suggesting interactions of CEACAM1-4S with the cytoskeleton were an important determinant of glandular morphogenesis. Interestingly, when mouse mammary carcinoma cells were grown in humanized NOD/SCID mouse mammary fat pads, only the 4L isoform initiated morphogenesis, the opposite of what was observed *in vitro* [21], raising questions about the equivalence of *in vitro* and *in vivo* models of morphogenesis.

Because of its role in cell adhesion, the CEACAM1 N-terminal Ig domain [22], [23], [24], like the cytoplasmic domain, has been the focus of numerous investigations. The adhesive epitope within the N-terminal Ig-domain has been defined for rat [24], mouse and human CEACAM1 [22], [23], the evolutionary relationships between CEACAM1 from different species has been determined [25], [26] and the three dimensional structure has been established by X-ray crystallography [27]. In comparison, the CEACAM1 transmembrane domain has received relatively little attention, perhaps because transmembrane domains have often been viewed as passive anchor sequences that span the lipid bilayer. Over the last 10 years, this simplistic viewpoint has fallen by the wayside in the face of accumulating evidence implicating transmembrane domains in helix-helix interactions leading to dimerization, oligomerization and signal transduction [28], [29], [30]. The possible involvement of transmembrane-transmembrane domain interactions in the functionality of CEACAM1 was suggested by the presence of repeating GXXXG motifs (where X represents any amino acid), sequences known to control protein dimerization and signaling [30], [31], and the presence of transmembrane C-terminal tyrosine residues shown in other proteins to be mediators of molecular recognition, self assembly and signal transduction [32]. In the present investigation, we have examined the effect of transmembrane domain mutations on the ability of CEACAM1-4S to confer an anchorage independent phenotype when expressed in a clonal line of CEACAM1 negative, anchorage dependent rat hepatocellular carcinoma cells, designated 253-NT. Our results show that transmembrane mutations in both GXXXG and tyrosine residues have both positive and negative effects on the anchorage independent phenotype produced by wild type CEACAM1-4S.

## Methods

### Antibodies

The origin and characteristics of MAb 5.4 specific for CEACAM1 and MAb 188.A2 specific for rat transferrin receptor have been described previously [33], [34]. Monoclonal antibody 9.2 (MAb 9.2) was provided by Drs. Werner Reutter and Oliver Baum at the Free University, Berlin, Germany [35]. Mouse anti-human HLA antibody was purchased from Sigma-Aldrich (Sigma-Aldrich Co., St. Louis, MO). The preparation of polyclonal rabbit anti-peptide antibodies specific for the CEACAM1-4L and CEACAM1-4S has been previously described [36]. The secondary antibodies used for indirect immunofluorescence labeling were Alexa-488 conjugated goat anti-mouse and goat anti-mouse-HRP conjugated secondary antibody (Invitrogen, Carlsbad, CA, USA).

### Cell Culture

The parental cell line 253T was established from a 2-acetylaminofluorene induced rat hepatocellular carcinoma, as described previously [35]. The anchorage dependent 253T-NT cell line was isolated from 253T by limiting dilution cloning. 253T and 253-NT cells were grown in Waymouth medium (Sigma, St. Louis, MO, USA) supplemented with 15% FBS, 1% glutamine (Invitrogen), 0.1% Gentamycin (Invitrogen), and 0.2% Normocin (Invivogen, San Diego, CA, USA). For cell proliferation assays, 1.5×10^4^ cells were plated in a 24-well plate. At 24, 48, 72, and 96 hours after plating, cells were trypsinized (Invitrogen), stained with trypan blue and counted using a hemocytometer.

### Construction of a CEACAM1-4S Expression Vector

RNA was isolated from a normal Fisher rat liver using RNAzol B according to the manufacturer's instructions (Tel-Test, Friendswood, TX). cDNA was synthesized from the purified RNA according to the manufacturer's instructions using the SuperScript III first-strand synthesis system for RT-PCR (Invitrogen). CEACAM1-4S cDNA was amplified by PCR from the total cDNA product using primers: CEACAM1-4S Forward 5′CAGGAATT-CATGGAGCTAGCCTCGGCT-3′ and CEACAM1-4S Reverse 5′-CGAGTCGACTCGTCAGAAGGAC CCAGATCC-3′. The primers contained *Eco*RI and *Sal*I restriction sites.

### Restriction Digest

A restriction digest was performed on both the CEACAM1-4S PCR product and the pCl-neo plasmid (Promega, Madison, WI, USA) using Eco*RI* and Sal*I* (New England Biolabs, Ipswich, MA, USA). Following digestion, the plasmid and the PCR product were dephosphorylated using Antarctic phosphatase (New England Biolabs), heat-treated to inactivate the phosphatase, and run on a 1% agarose gel. Bands corresponding to the plasmid DNA and CEACAM1-4S PCR product were purified using the Geneclean spin kit (Qbiogene, Morgan Irvine, CA, USA). The CEACAM1-4S PCR product was ligated into the pCI-neo plasmid using T4 DNA ligase (New England Biolabs). Ligated plasmid was transformed into One Shot OmniMax 2 T1 Phage-Resistant Cells (Invitrogen) according to the manufacturer's protocol. Transformed cells were plated onto LB/CARB plates and resulting colonies were screened by PCR using forward and reverse primers for CEACAM1-4S (see above). Plasmid DNA from four CEACAM1-4S positive clones was isolated using the Qiagen Endofree maxiprep kit (Valencia, CA, USA) and submitted for DNA sequencing to the W.M. Keck Biotechnology Laboratory at Yale University (New Haven, CT).

### Selection of Sites for Mutagenesis

Mutation sites were chosen based on the consensus location of the transmembrane domain predicted by the different membrane topology algorithms shown in Table 1. The results from this analysis showed that the various transmembrane prediction algorithms delineated transmembrane domains with variable N (G424 to I431) and C (Y445 to S449) termini and different numbers of potential GXXXG motifs. All of the predicted transmembrane domains lacked the terminal GLSE predicted by Kyte Doolittle and Hopp-Wood (Table 1) and thus did not include the G420-LSE-G424 motif. Ten of the 12 predicted transmembrane domains included G428, strongly suggesting the presence of the two motifs centered on G432 (G428-IVI-G432 and G432-SVA-G436). Although G424 was present in only 3 of 12 predicted TM, G424's location at the N-terminus of a tandem array of three classic GXXXG motifs caused us to consider that the G424-AIA-G432 sequence might function as a dimerization motif when all the other GXXXG motifs were disrupted by G428L and G436L mutations. With regard to the C terminus, all of the predicted transmembrane domains contained Y445 and 7 of 12 contained Y448, leading us to conclude with reasonable certainty that Y448 was within the transmembrane domain or at the very least, at the interface between the transmembrane and cytoplasmic domains. Taking all of these considerations into account, we arrived at the transmembrane sequence proposed in Table 2. The brackets denote the uncertainty with regard to G424 and Y448. In recognition of the variability in the predicted N-terminus and the uncertainty regarding the functionality of the G424-AIA-G428 motif, we introduced a G424L mutation to disrupt this motif and a G432L mutation to knock out both the G428-IVI-G432 and the G432-SVA-G436 motifs. Disruption of all three of the possible GXXXG motifs was accomplished by introducing both G424L and G432L mutations. To disrupt tyrosine mediated interactions, Y445 and Y448 were replaced with V, the choice of a tyrosine to valine mutation being based on the following considerations: 1) valine lacks a hydroxy group; 2) valine does not have an aromatic side chain; 3) valine is non-polar and thus should not disrupt the alpha helix or alter transport to the plasma membrane.

**Table 1:** Survey of Servers.

Amino Acid Sequence:	Source
GLSE**G**AIAGIVI**G**SVAGVALIAALA**Y**FL**Y**	Wild type
424 432 445 448	
GLSE**G**AIAGIVI**G**SVAGVALIAALA**Y**FL**Y**	FASTA KYTE-DOOLITTLE^1^
SGLSE**G**AIAGIVI**G**SVAGVALIAALA**Y**FL**Y**S	ProtScale Hopp-Woods^2^
**G**AIAGIVI**G**SVAGVALIAALA**Y**F	SOSUI^3^
IAGIVI**G**SVAGVALIAALA**Y**FL**Y**	TMHMM2.0^4^
IAGIVI**G**SVAGVALIAALA**Y**F	PREDICT PROTEIN (PHDhtm)^5^
AIAGIVI**G**SVAGVALIAALA**Y**FL**Y**	PREDICT PROTEIN (solvent access)^6^
AIAGIVI**G**SVAGVALIAALA**Y**FL	HMMTOP; DAS^7^
IVI**G**SVAGVALIAALA**Y**FL**Y**S	TMpred^8^
I**G**SVAGVALIAALA**Y**FL**Y**SRKTG	PALEALE^9^
AIAGIVI**G**SVAGVALIAALA**Y**	OCTOPUS^10^
IAGIVI**G**SVAGVALIAALA**Y**FL**Y**	WESA^11^
GIVI**G**SVAGVALIAALA**Y**FL	MEMSAT3^12^

1 http://fasta.bioch.virginia.edu/fasta_www2/fasta_www.cgi,

2 http://web.expasy.org/cgi-bin/protscale/protscale.pl,

3 http://bp.nuap.nagoya-u.ac.jp/sosui/sosui_submit.html,

4 http://www.cbs.dtu.dk/services/TMHMM/,

5 https://www.predictprotein.org/,

6 http://www.enzim.hu/hmmtop/html/submit.html,

7 http://www.sbc.su.se/~miklos/DAS/,

8 http://www.ch.embnet.org/software/TMPRED_form.html,

9 http://distill.ucd.ie/paleale/,

10 http://octopus.cbr.su.se,

11 http://pipe.scs.fsu.edu/wesa.html,

12 http://bioinf.cs.ucl.ac.uk/psipred/.

**Table 2:** CEACAM1-4s Transmembrane Domain.

	Amino Acid Sequence:
Wild type	GLSE**[G]**AIAGIVI**G**SVAGVALIAALA**Y**FL**[Y]**
Residues of Interest	424 432 445 448

Mutants:	
G424L	GLSE**[L]**AIAGIVIGSVAGVALIAALAYFL[Y]
G432L	GLSE[G]AIAGIVI**L**SVAGVALIAALAYFL[Y]
G424L/G432L	GLSE**[L]**AIAGIVI**L**SVAGVALIAALAYFL[Y]
Y445V	GLSE[G]AIAGIVIGSVAGVALIAALA**V**FL[Y]
Y448V	GLSE[G]AIAGIVIGSVAGVALIAALAYFL**[V]**
Y445V/Y448V	GLSE[G]AIAGIVIGSVAGVALIAALA**V**FL**[V]**
G424L/G432L/Y445V	GLSE**[L]**AIAGIVI**L**SVAGVALIAALA**V**FL[Y]
G424L/G432L/Y448V	GLSE**[L]**AIAGIVI**L**SVAGVALIAALAYFL**[V]**
G424L/G432L/Y445V/Y448V	GLSE**[L]**AIAGIVI**L**SVAGVALIAALA**V**FL**[V]**

### Site-directed Mutagenesis

Site-directed mutagenesis was performed on the pCI-neo plasmid containing the CEACAM1-4S gene using the QuikChange II XL Site-directed mutagenesis kit (Stratagene, La Jolla, CA, USA) according to the manufacturer's protocol. The oligonucleotide primers used for mutagenesis were as follows: G424L Forward 5′-CTGGCCTCTCAGAGCTTGCCATTGCAGGCATTGTG-3′, G424L Reverse 5′-CACAATGCCTGCAATGGCAAGCTCTGAGAGGCCAG-3′, G4-31L Forward 5′-GCAG-GCATTGTGATTCTATCTGTGGCTGGAGTGGCC-3′, G432L Reverse 5′-GGCCACTCCAGCCACA-GATAGAATCACAATGCCTGC-3′, Y448V Forward 5′-GCGCTGGCATACTTCCTTGTTTCCAGGA-AGACTGGC-3′, Y448V Reverse 5′-GCCAGTCTTCCTGGAAACAAGGAAGTATGCCAGCGC-3′, Y445V Forward 5′-CCTAATAGCAGCGCTGGCAGTCTTCCTTTATTCCAGG-3′, Y445V Reverse 5′-CCTGGAATAAAGGAAGACTGCCAGCGCTGCTATTAGG-3′. To create the Y445V and Y448V double mutant, a Y448V mutation was introduced into the Y445V mutant using the following primer set: Forward 5′-GCGCTGGCAGTCTTCCTTGTTTCCAGGAAGACTGGC-3′; Reverse 5′-GCCAGTCTTC-CTGGAAACAAGGAAGACTGCCAGCGC-3′.

### Generation of Stably Transfected Sublines of 253T-NT

For transfection of 253-NT cells, 1×10^6^ cells at 75% confluence were transfected with 7.5 µg of plasmid DNA in Lipofectamine LTX reagent (Invitrogen) following the manufacturer's recommended protocol. After incubation for 24 hours in complete medium, stable transfectants were selected by maintaining cells in complete medium containing geneticin (Invitrogen) at 600 µg/ml with medium changes every 48 hours. For all subsequent experiments, geneticin resistant cells were maintained in complete selection medium.

### Indirect Immunofluorescence

Cells were seeded in permanox chamber slides (Nalge Nunc International, Rochester, NY) at a density of 1×10^5^ cells/ml and incubated in a 5% CO_2_ humidified chamber for 48–72 hours. Cells were washed three times in ice-cold PBS and fixed for 10 min in ice-cold acetone. Normal liver sections and frozen tumor sections were prepared as previously described [37]. Cells were blocked for 15 min with 1% normal goat serum (Sigma-Aldrich) in PBS and incubated for 30 min at room temperature with a 1∶200 dilution of primary MAb 9.2 specific for CEACAM1-4S. After three washes in PBS, cultures were fixed with 4% paraformaldehyde for 1 min, quenched in 0.1 M glycine in PBS, and incubated at 4°C for 30 min in Alexa 488 conjugated goat anti-mouse IgG (1∶400 dilution). Cells were examined by fluorescence microscopy using a Nikon Eclipse E800 microscope (Nikon Instruments, Inc., Melville, NY).

### Confocal Microscopy

Confocal images were acquired with a Nikon PCM 2000 (Nikon Inc. Mellville, NY, USA) using the Argon (488) and the green Helium-Neon (543) lasers. Serial optical sectioning was performed with Simple 32, C-imaging computer software (Compix Inc, Cranberry Township, PA, USA). Z series sections were collected at 0.5 mm using a 60× PlanApo objective and a scan zoom of 2×. Images were processed in NIH Image J (National Institutes of Health, Springfield, VA, USA).

### FACS Analysis

Cells were harvested at 75% confluence using non-enzymatic cell dissociation solution (Sigma-Aldrich) and labeled in suspension with anti-CEACAM1-4S monoclonal antibody MAb 9.2 as described above. Primary and secondary antibodies were diluted in 1% normal goat serum in PBS and incubated sequentially with cells at 4°C for 20 min. Cells were washed with sterile ice cold sort buffer (Ca/Mg++ free, pH 7.0 PBS, 5 mM EDTA, 2 mM HEPES and 1% FBS) and resuspended at a final concentration of 8–10×10^6^ cells/ml. CEACAM1-4S positive cells were isolated by FACS as previously described by Comegys et al. [38].

### Anchorage Independent Growth

Cells were harvested using non-enzymatic cell dissociation solution (Sigma-Aldrich), suspended in 0.76% low melting point agar diluted 1∶1 with 2× complete Waymouth medium (Sigma-Aldrich) and seeded in 6 well plates (10^4^ cells/well) coated with 2 mls of 1.25% low melting point agar (Sigma-Aldrich) diluted 1∶1 with 2× complete Waymouth medium. Plates were incubated at 37°C for three weeks and analyzed microscopically to identify anchorage independent clones. Colony size was determined from digital micrographs using Image-Pro Plus 5.0 software (Media Cybernetics, Inc., Bethesda, MD) to determine the average area calculated from measurements made on a total of 15–30 colonies.

### Tumorigenicity in Nude Mice

All animal studies were performed using protocols approved by the Rhode Island Hospital Institutional Animal Care and Use Committee (cmtt# 0268-98). Athymic nude mice were purchased from Harlan (Indianapolis, IN). Twenty-four hours prior to transplantation of tumor cells, animals were injected intraperitoneally with anti-LY2.2 antibodies containing anti-asialo GM1 to suppress T-cell and NK cytotoxic activity, respectively [39], [40]. Cultured cells were harvested as described previously [13]. Subcutaneous injections with 5×10^6^ cells per site were performed under aseptic conditions into both of the upper flanks. For intraperitoneal injections, mice were injected with 2×10^6^ cells suspended in HBSS. Tumors were excised at three weeks post-injection, weighed, frozen in a hexane/acetone bath and stored at −80°C. Tumors were analyzed by indirect immunofluorescence as described above.

### Immunoblot Analysis Following Blue-Native (BN) PAGE

BN-PAGE analysis was performed on crude membrane isolates prepared from 253T-NT cells stably transfected with empty vector, wild type CEACAM1-4S and each of the single and double glycine mutants described above. Cell pellets were thawed on ice and lysed following 10 passages through a 20-gauge needle. Lysates were centrifuged at 3,500 rpm for 15 min at 4°C and MgCl_2_ and benzonase (Sigma Aldrich) were added to the supernatant to give final concentrations of 2 mM and 1 unit/µl, respectively. After incubation at room temperature for 30 min, membranes were centrifuged at 17,000 rpm for 15 min at 4°C. Blue-native (BN) PAGE electrophoresis was performed using the Native Page Novex Bis-Tris gel system (Invitrogen). Membrane pellets were solubilized on ice for 30 min in 1× Native Page sample buffer (Invitrogen) containing 1% dodecyl-ß-D-maltoside (Invitrogen) and protease inhibitors. After a 30 min incubation, the samples were centrifuged at 20,000× g for 20 min at 4°C to remove insoluble material. A Bradford assay (Bio-Rad, Hercules, CA, USA) was performed to determine protein concentration. One ml of 5% NativePage G-250 sample additive (Invitrogen) was added to each sample, and the samples and a Native Mark protein ladder (Invitrogen) were loaded into the wells of a 4–16% Bis-Tris gel (Invitrogen). After running at 150 V for approximately 30 min, the cathode buffer was changed from 0.02% to 0.002% G-250 and electrophoresis at 150 V was continued for an additional 120 min. BN-PAGE gels were immunoblotted onto PVDF membranes (Bio-Rad). Transfer was performed using Bio-Rad's semi-dry transfer apparatus for 1 hour at 25 V. After transfer, membranes were incubated in 8% acetic acid for 15 min, rinsed with water and air-dried. PVDF membranes were re-hydrated with methanol, rinsed with water and blocked in 5% milk overnight at 4°C. Blots were labeled with antibodies as previously described [24].

### Immunoblot Analysis Following SDS-PAGE

Immunoblots were prepared with extracts from 80% confluent cell cultures lysed in RIPA buffer (Pierce, Rockford, IL, USA) containing protease and phosphates inhibitors (Calbiochem). Cell lysates were centrifuged at 14,000 rpm for 15 min and resolved by SDS-PAGE. Immunoblots were prepared and visualized as previously described, using MAb 9.2 to detect CEACAM1-4S [13]. The density of the MAb 9.2 reactive bands was determined by image analysis of digital images of immunoblots captured using a Versadoc Imaging System (Bio-Rad) and Quantity One software (Bio-Rad), as previously described [13].

### Statistical Analysis

A paired t-test was performed to determine statistical significance using GraphPad QuickCalcs software (GraphPad Software Inc., La Jolla, CA).

## Results

### Expression of CEACAM1-4S by 253-NT Cells Restores the Anchorage Independent Phenotype Exhibited by the Parental 253T Cell Line

**Figure 1:**
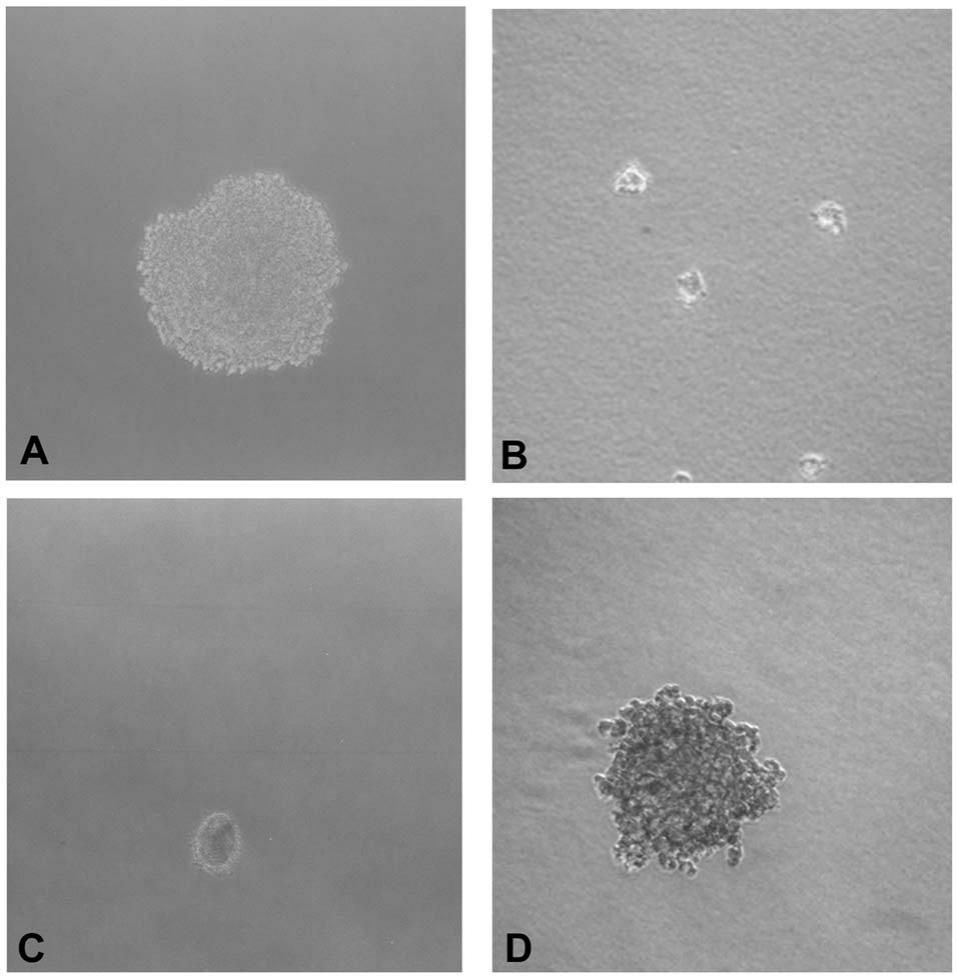
Anchorage Independent Growth Induced by Expression of CEACAM1-4S. (**A**) A representative anchorage independent colony formed by parental 253T cells after 3 weeks in soft agar. (**B**) Sparsely scattered single cells that typified soft agar cultures of 253T-NT cells. (**C**) Scattered cells remaining after 3 weeks in soft agar cultures of 253T-NT cells stably transfected with empty vector. (**D**) A representative colony formed by253T-NT cells stably transfected with a wild type CEACAM1-4S expression vector.

In previous investigations, we focused on the structural and functional determinants of tumor suppression mediated by CEACAM1-4L, one of two major splice variants expressed in rat liver [13], [16]. The primary goal in these studies was to gain insight into the role of the cytoplasmic and extracellular domains in cell adhesion and tumor suppression. In the present report, we have focused on the CEACAM1 transmembrane domain, a relatively uncharacterized region shared by both the 4L and 4S isoforms. Our cell model for these studies was an anchorage dependent, CEACAM1 negative subclone designated 253-NT that was derived clonally from the parental rat 253T cell line [6]. When suspended in soft agar, 253T cells continued to grow and by three weeks had formed well-defined anchorage independent colonies (Figure 1A). In comparison, soft agar cultures of 253-NT cells showed no evidence of significant growth and after three weeks were composed almost entirely of single cells (Figure 1B). In contrast, 253-NT cells stably transfected with a wild type CEACAM1-4S expression plasmid and enriched by FACS to yield cultures at least 70% positive for CEACAM1-4S expression regained the anchorage independent phenotype of the parental 253T cells (Figure 1C) while cells stably transfected with empty vector (Figure 1D) had not grown significantly after 3 weeks in soft agar.

### Wild Type and Transmembrane Domain Mutants were Expressed at Similar Levels on the Surface of Stably Transfected 253T-NT Cells

Sequence analysis showed that the CEACAM1-4S transmembrane domain contained multiple GXXXG dimerization motifs and two C-terminal tyrosine residues which in other transmembrane receptors were involved in transmembrane domain dependent signaling events [30], [31], [32]. To determine if these transmembrane domain elements played a part in the anchorage independent phenotype induced by CEACAM1-4S, nine expression vectors encoding CEACAM1-4S with transmembrane domain mutations in the GXXXG motifs and/or tyrosine residues were constructed as described under Methods and shown in Table 1. These vectors were used to produce stably transfected sublines of 253-NT. Single G424L or G432L mutations or double G424L/G432L mutations were introduced to disrupt two or more GXXXG motifs. Substituting leucine for glycine introduced a large hydrophobic amino acid that maintained the hydrophobic character of the transmembrane domain but disrupted the conformation of the transmembrane domain helix [28]. Transmembrane domain tyrosine residues were mutated to valine, a substitution that replaced tyrosine with a non-polar hydrophobic amino acid lacking a hydroxyl group in its side chain. In other systems, proper spacing of the tyrosine hydroxyl group had been shown to be necessary for signaling events dependent upon protein-protein interactions [41].

**Figure 2:**
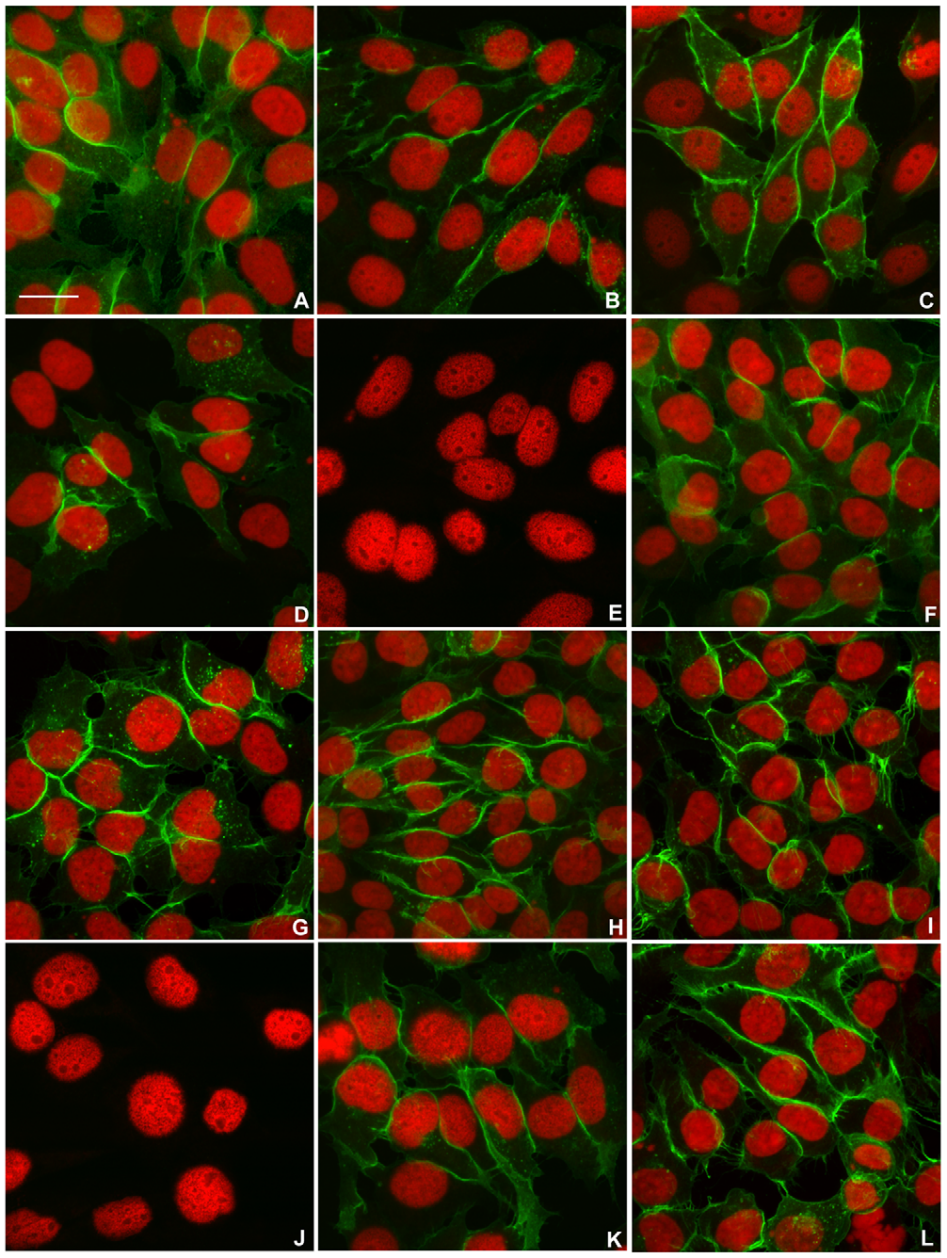
Cell Surface Expression of CEACAM1-4S. 253T-NT cultures stably transfected with wild type or mutated forms of CEACAM1-4S were labeled by indirect immunofluorescence with MAb 9.2, a monoclonal antibody specific for CEACAM1. Cell nuclei were stained with propidium iodide. Confocal digital images constructed from 7–15 optical sections for each labeled subline are shown in panels A–L. (**A**) wild type CEACAM1-4S; (**B**) G424L mutant; (**C**) G432L mutant; (**D**) G424L and G432L double mutant; (**E**) Empty vector; (**F**) Y445V mutant; (**G**) Y448V mutant; (**H**) Y445V and Y448V double mutant; (**I**) G424L, G432L, Y445V, Y448V quadruple mutant; (**J**) untransfected 253T-NT cells; (**K**) G424L, G432L and Y445V triple mutant; (**L**) G424L, G432L and Y448V triple mutant. Scale bar represents 20 mm.

**Figure 3:**
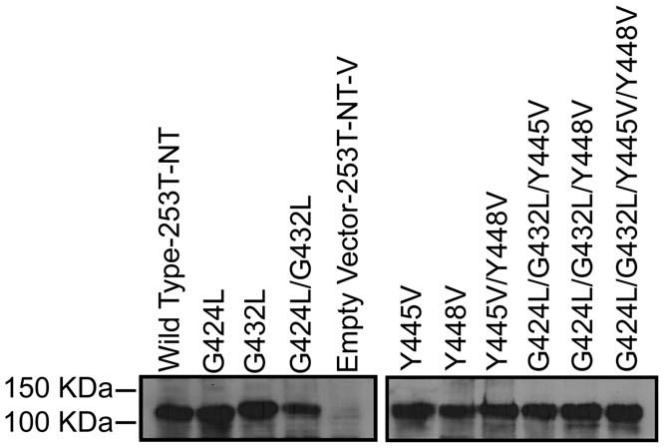
Immunoblot Analysis Shows that Wild Type and Mutant CEACAM1-4S Constructs Have Identical Molecular Mass. Protein lysates prepared from wild type cells and each of the 253T-NT transfected cell lines were resolved on 7.5% SDS-polyacylamide gels, transferred onto nitrocellulose and labeled with MAb 9.2 specific to CEACAM1. Analysis of expressed wild type and mutant CEACAM1-4S protein shows that reactive band corresponding to CEACAM1-4S had the same apparent molecular mass (105 kDa).

**Figure 4:**
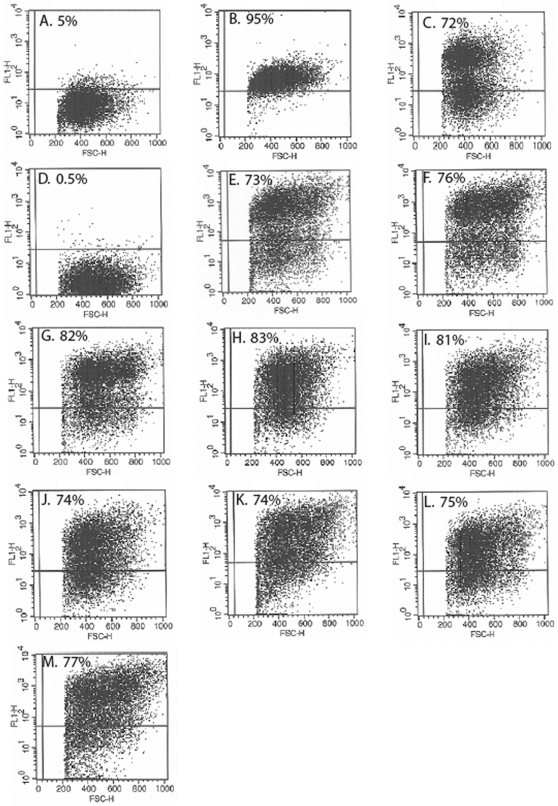
FACS Analysis of CEACAM1-4S Expression in Transfected and Untransfected 253T-NT Sublines. FACS analysis was performed to determine the percentage of CEACAM1-4S positive cells in the various sublines. When 253T-NT cells were labeled with a human HLA specific monoclonal antibody (negative control), only 5% of the cells showed fluorescence above background levels (**A**). When stained with MAb 188-A2 specific for transferrin receptor (positive control), 95% of the cells showed positive reactivity (**B**). (**C–M**) the percentage of positive cells is indicated in the upper left corner of each histogram. Sublines analyzed in each panel were transfected with: (**C**) wild type CEACAM1-4S; (**D**) empty vector; (**E**) G424L; (**F**) G432L; (**G**) G424L and G432L; (**H**) Y445V; (**I**) Y448V; (**J**) Y445V and Y448V; (**K**) G424L, G432L and Y445V; (**L**) G424L, G432L and Y448V; (**M**) G424L, G432L, Y445V and Y448V.

Examination by confocal fluorescence microscopy of cultures established from CEACAM1-4S positive cells isolated by FACS showed intense membrane fluorescence when labeled by indirect immunofluorescence with CEACAM1 specific MAb 9.2, confirming that both wild type and mutant forms of CEACAM1-4S were properly transported to the plasma membrane (Figure 2, A–L). Empty vector transfected and untransfected 253-NT cells showed no detectable reactivity with MAb 9.2 (Figure 2, E and J, respectively). Immunoblot analysis indicated that the wild type and mutant proteins all had an apparent molecular mass of 105 kDa, the expected size for CEACAM1-4S isoform [2], suggesting that post-translational processing has not been altered (Figure 3). Quantitative analysis by flow cytometry of FACS selected, stably transfected cells labeled by IIF with MAb 9.2 indicated that 72–83% of the cells were positive for either wild type or mutated forms of CEACAM1-4S (Figure 4).

### Soft Agar Colonies of 253T-NT Cells Expressing Y to V Mutants Increased in Size at a Much Faster Rate Than Colonies Composed of Cells Transfected with WT-CEACAM1-4S or G to L Mutants

**Figure 5:**
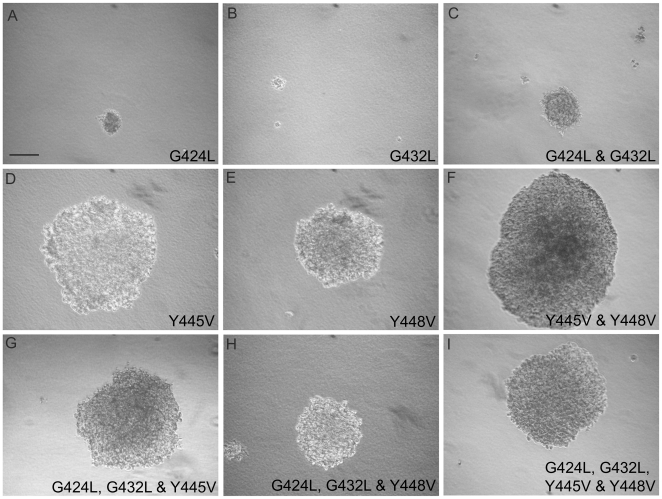
Anchorage Independent Growth in Soft Agar. (**A–I**) show the appearance of colonies formed by the various CEACAM1-4S sublines after 3 weeks in soft agar: (**A**) G424L; (**B**) G432L; (**C**) G424L and G432L; (**D**) Y445V; (**E**) Y448V; (**F**) Y445V and Y448V; (**G**) G424L, G432L and Y445V; (**H**) G424L, G432L and Y448V; (**I**) G424L, G432L, Y445V and Y448V.

**Figure 6:**
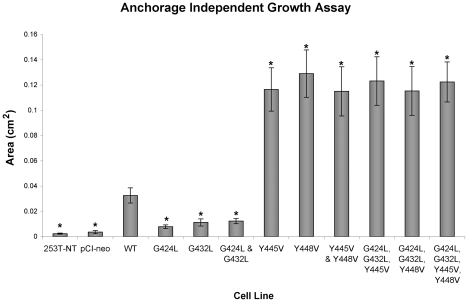
Size Distribution of Soft Agar Colonies. Image ProPlus software was used to calculate the average area of soft agar colonies. The results represent three separate soft agar growth experiments. The columns marked with an asterisk indicate sublines that were either significantly smaller or larger than those formed by cells transfected with wild type (WT) CEACAM1-4S (p<0.05).

The effect of transmembrane domain mutations on anchorage independent growth was determined from changes in the average areas of soft agar colonies (Figure 5 and 6). When 253T-NT cells expressing wild type (Figure 1C), single or double tyrosine mutants of CEACAM1-4S were grown in soft agar, sublines expressing tyrosine mutants (Figure 5 D, E, F and Figure 6) showed a 3.5-fold increase in colony size relative to cells transfected with the wild type protein (Figure 1C and Figure 6). In contrast, 253T-NT cells expressing CEACAM1-4S with GXXXG motifs disrupted by G to L mutations (Figure 5A, B, C and Figure 6) either did not grow or formed colonies that for the double glycine mutant (Figure 5C and 6), were less than half the size of those formed by cells expressing the wild type protein (Figure 1C and Figure 6). Moreover, the rapid growth phenotype conferred by tyrosine to valine mutants appeared to be dominant over the growth suppressed phenotype displayed by glycine to leucine mutants since all of the sublines with both glycine to leucine and tyrosine to valine mutations displayed enhanced anchorage independent growth (Figure 5G, H, I and Figure 6).

### The Size of Soft Agar Colonies Formed by 253T-NT Cells Expressing Wild Type and Mutant Forms of CEACAM1-4S was Closely Correlated with the Rate of Proliferation

**Figure 7:**
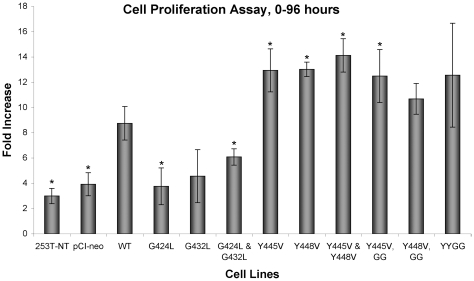
Rates of Cell Proliferation of CEACAM1-4S Transfectants. The bars show the fold-increase in cells at 96 hours after plating. The columns marked with an asterisk were sublines showing an increase in cell number between 0 and 96 hours that according to P values (p<0.05), was significantly higher or lower than 253T-NT cells transfected with wild type (WT) CEACAM1-4S. In general, the number of 253T-NT cells expressing G to L mutants increased at a slower rate and Y to V mutants at a higher rate than cells expressing wild type CEACAM1-4S. Results shown represent four separate assays.

Proliferation assays were carried out to ascertain whether the size of soft agar colonies (Figure 5 and 6) was proportional to the rate of proliferation *in vitro* calculated from changes in cell number as a function of time. As shown in Figure 7, 253T-NT cells expressing wild type CEACAM1-4S proliferated 2.2 times faster than 253T-NT cells carrying the empty vector and from 1.4-2.2 times faster than cells transfected with glycine mutants. Consistent with their rapid growth in soft agar, 253T-NT cells expressing CEACAM1-4S with single or double tyrosine mutants proliferated at rates that were 1.4–1.6-fold higher than cells with wild type CEACAM1-4S. Taken together, these data suggested that the CEACAM1-4S transmembrane domain was controlling interactions involved in growth under anchorage independent conditions, interactions that were altered by GXXXG or tyrosine mutations.

### Transmembrane Mutations Change the Ability of Wild Type CEACAM1-4S to Promote a Tumorigenic Phenotype in Nude Mice

**Figure 8:**
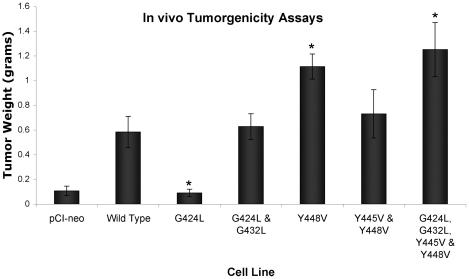
Tumorigenicity of 253T-NT Transfected with WT and Mutated CEACAM1-4S. Three nude mice were injected in the front flanks with each subline of 253T-NT (6 injection sites for each cell line). At three weeks after injection, tumor nodules were harvested and weighed. Columns marked with an asterisk indicate cell lines that according to P values (p<0.05) were significantly larger (Y448V and the quadruple mutant) or smaller (G424L) than 253T-NT cells expressing wild type CEACAM1-4S. Although the effects of mutations were not as clear-cut as those observed for growth in soft agar, the G424L mutation compromised and the Y448V or the quadruple mutation enhanced the ability of CEACAM1-4S to produce tumors in nude mice.

**Figure 9:**
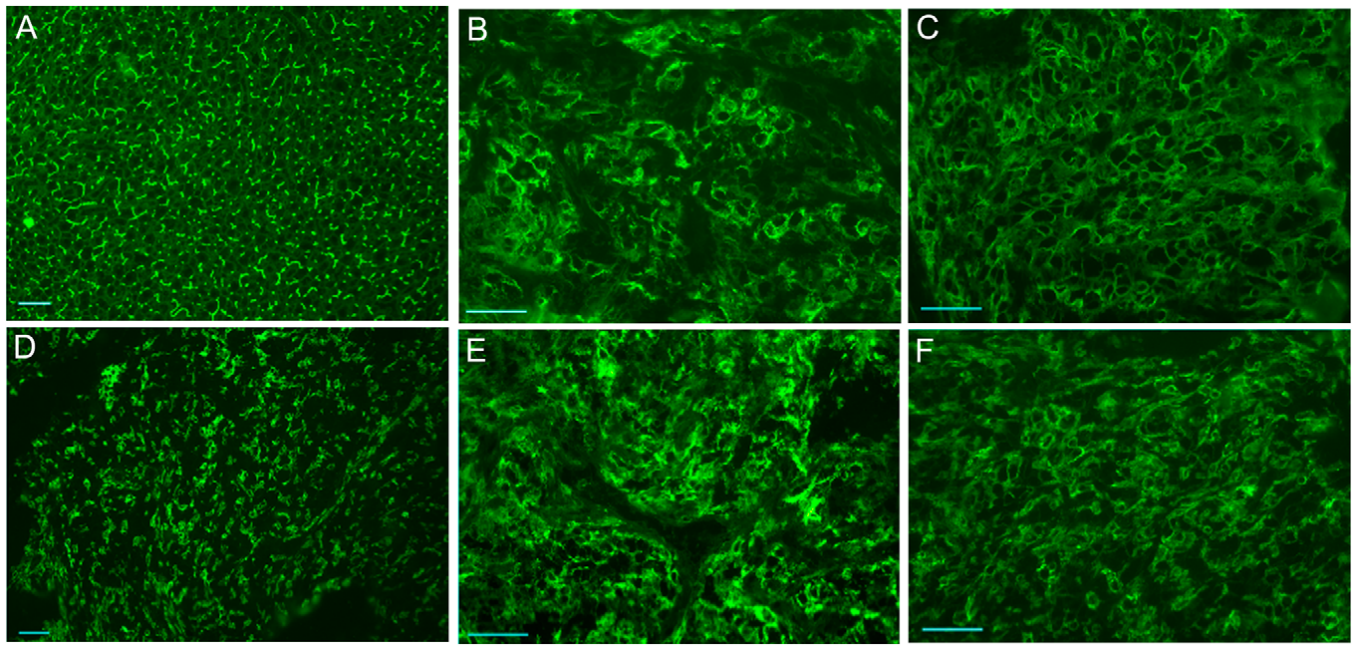
Expression of WT and Mutant CEACAM1-4S on Tumor Nodules. Frozen sections of normal liver or 253T-NT transfectants were stained by indirect immunofluorescence with MAbs 9.2 and 5.4, monoclonal antibodies recognizing the N-terminal Ig domain of CEACAM1-4S. (**A**) Normal rat liver with MAb 5.4; (**B**) 253T-NT expressing wild type CEACAM1-4S; (**C–F**) tumors of 253-NT cells expressing the following mutants: (**C**) G424L with MAb 9.2; (**D**) G424L and G432L with MAb 5.4; (**E**) Y448V with MAb 5.4; (**F**) Y445V and Y448V with MAb 9.2.

To determine if changes in anchorage independent growth induced by transmembrane domain mutations were mirrored by altered tumorigenicity, nude mice were injected subcutaneously in the upper flanks with 253T-NT cell lines expressing wild type or mutant CEACAM1-4S. Subcutaneous tumors were harvested from animals at three weeks after injection, a time point chosen by necessity because of the large tumor burden in animals injected with 253T-NT cells expressing tyrosine mutants. On average, tumors produced by cells expressing wild type CEACAM1-4S were 5.5- and 6.5-fold larger by weight than those generated by cells transfected, respectively, with the empty vector or the single G424L mutant (Figure 8). While cells expressing the wild type protein formed tumors comparable in weight to those formed by cells expressing the double G to L mutant, 253T-NT cells expressing CEACAM1-4S with single Y448V, double Y445V/Y448V or quadruple G424L/G432L/Y445V/Y448V mutations produced tumors that were 1.9-, 1.25-, and 2.14-fold larger than those produced by cells transfected with the wild type protein (Figure 8). Indirect immunofluorescence analysis of frozen tumor sections, confirmed that tumor nodules formed by 253-NT cells transfected with either wild type or transmembrane domain mutants remained strongly positive for CEACAM1-4S (Figure 9).

### Disruption of GXXXG Motifs by Double G to L Mutations Caused an Increase in the Mobility of Wild Type CEACAM1-4S Detected in Western Blots Prepared from Blue Native Gels

**Figure 10:**
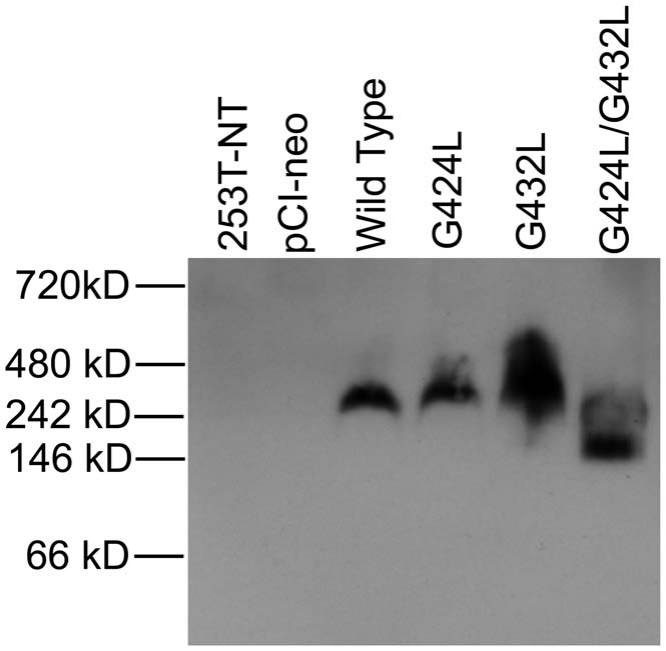
Mobility of G to L Mutants of CEACAM1-4S Resolved by BN-PAGE. Protein lysates were prepared and resolved by BN-PAGE as described under materials and methods. Proteins were transferred onto PVDF membranes and probed with monoclonal antibody 9.2. When separated on native gels, wild type CEACAM1-4S and the single G mutants migrated with an apparent molecular mass that was approximately 100 kDa higher than the double glycine mutant.

Previous reports have demonstrated that dimerization via transmembrane domain helix-helix interactions are often mediated by GXXXG or GXXXA motifs within the transmembrane domain [30], [42]. To determine if the GXXXG motifs within the transmembrane domain of CEACAM1-4S played a role in the dimerization of CEACAM1-4S [36], [43], the effect of G to L mutations on CEACAM1-4S interactions was analyzed by Blue-Native polyacrylamide gel electrophoresis (BN-PAGE). In BN-PAGE, Coomassie G-250 is used in place of SDS to coat proteins with a uniform negative charge without causing denaturation or disruption of protein-protein interactions [44]. As shown in Figure 10, wild type and single G-to-L mutants of CEACAM1 demonstrated an apparent molecular mass by BN-PAGE that was approximately 100 kDa higher than the double G to L mutant, a difference approximately the size of CEACAM1-4S resolved by reducing SDS-PAGE. These data suggested that a single GXXXG motif was sufficient to mediate dimerization of CEACAM1-4S and/or interaction with another yet-to-be identified transmembrane protein, interactions that appeared to require helix-helix interactions mediated by GXXXG motifs.

## Discussion

The ability of CEACAM1-4S expression in the context of the proteome of 253-NT cells to restore the tumorigenic and anchorage independent growth characteristics of the parental 253T cell line [6] provided a quantifiable, reproducible endpoint for examining the functionality of the CEACAM1-4S transmembrane domain. The idea that CEACAM1 phenotypes could be context specific came from our previous studies showing that CEACAM1-4L expression dramatically suppressed the tumorigenicity of CEACAM1 negative PC-3 human prostate carcinoma cells [13]. With continued passage, however, cells eventually reacquired a tumorigenic phenotype without losing expression of CEACAM1-4L, suggesting a strong selection for cells with proteomes that were unable to support CEACAM1 mediated tumor suppression. Context dependent effects on CEACAM1-4S phenotypes were also suggested by the ability of CEACAM1-4S to induce morphogenesis of MCF7 cells *in vitro* but not *in vivo* [21].

Analysis of the amino acid sequence of the CEACAM1-4S transmembrane domain revealed the presence of four GXXXG sequences (Table 1 and 2), a motif known to drive high affinity helix-helix interactions that stabilize the dimerization/oligomerization of many well characterized proteins such as glycophorin A, epidermal growth factor receptor and the G protein-coupled α-factor receptor of budding yeast [30], [42], [45]. For the latter protein, disruption of the GXXXG motif not only impaired oligomerization but also disrupted signaling [30], [46]. Previous investigations have shown that both the long and short isoforms of CEACAM1 form *cis*-dimers [36], [43], an interaction that we hypothesized should be stabilized by GXXXG mediated helix-helix associations. To test this idea, we introduced glycine-to-leucine mutations that disrupted one or more transmembrane domain GXXXG motifs. FACS, fluorescent confocal microscopic and immunoblot analysis of cells stably transfected with the glycine-to-leucine mutants confirmed that the mutant proteins were expressed on the cell surface at the same level and the same size as wild type CEACAM1-4S. However, after 3 weeks in soft agar, cells expressing single glycine mutants showed significantly lower rates of proliferation and significantly smaller colonies when compared to cells transfected with wild type CEACAM1-4S, suggesting that a single glycine-to-leucine mutation had compromised the ability of CEACAM1-4S to induce anchorage independent growth. From these results, we concluded that the smaller size of colonies produced by glycine mutants resulted at least in part from a decrease in the rate of proliferation.

Since the double glycine mutation disrupted all of the GXXXG motifs, a corresponding decrease in either homo- or hetero- *cis*-dimerization would be expected if these motifs were the sole mediators of this interaction. However, impaired dimerization could also occur if a reduction in GXXXG motifs destabilized *cis*-dimers formed by single glycine mutants with a subsequent reduction in their steady state levels. Blue native-PAGE analysis showed that the apparent size of the double but not the single glycine mutants showed approximately a 100 kDA decrease in size, a shift consistent with impaired dimerization. Moreover, since single glycine to leucine mutations produced the growth suppressed phenotype but only double mutations hampered dimerization, it was concluded that even without an apparent effect on dimerization, glycine to leucine point mutations in the transmembrane domain had a profound effect on the growth phenotype produced by CEACAM1-4S.

With one exception, the effects of transmembrane domain mutations on growth *in vivo* were similar to those observed *in vitro*. In keeping with their limited capacity for anchorage independent growth, cells transfected with empty vector were poorly tumorigenic relative to cells expressing the wild type CEACAM1-4S. As predicted from their growth in soft agar, cells expressing the G424L mutant formed tumors considerably smaller than those derived from cells expressing wild type CEACAM1-4S. However, the similarity in the size of tumors generated by cells expressing the wild type, double glycine and double tyrosine mutants was at odds with *in vitro* assays where cells expressing wild type CEACAM1-4S showed a significantly greater or lesser capacity, respectively, for anchorage independent growth than cells expressing double glycine or tyrosine mutants. This discrepancy is reminiscent of the differential in glandular morphogenesis exhibited *in vitro* and *in vivo* by MCF7 mammary carcinoma cells [21] and thus could reflect differential effects of the subcutaneous microenvironment.

In general, tumors formed by cells expressing the single and double tyrosine mutants were much larger than those generated by cells transfected with wild type CEACAM1-4S or empty vector, a trend consistent with the greater rate of proliferation shown *in vitro* by tyrosine mutants. When viewed as a whole, these findings show that the phenotypes manifested *in vitro* by cells expressing transmembrane domain mutants are recapitulated *in vivo*, suggesting these changes are intrinsic to the transmembrane domain and are not the result of extrinsic factors e.g., microenvironment, that change the phenotypic effects of CEACAM1-4S by altering its proteomic context.

The decrease in soft agar growth produced by glycine mutations and the increase by tyrosine mutations relative to the wild type CEACAM1-4S would classify these, respectively, as loss-of-function and gain-of-function mutations [47], [48], [49]. Gain-of-function mutations are usually dominant, a characteristic apparent for the glycine/tyrosine mutants of CEACAM1-4S where the growth enhanced phenotype of tyrosine mutants was dominant over the growth inhibited phenotype resulting from glycine mutations. Based on the known functions of GXXXG motifs, it seems likely that the loss-of-function caused by glycine mutations is related to changes in helix-helix interactions that destabilize rather than prevent the formation of cis-dimers [28], [50], [51], a possibility consistent with the fact that single G424L or G432L mutations failed to disrupt dimerization (Figure 10) but did cause a decrease in anchorage independent growth. Also noteworthy is the marked decrease in cis-dimerization (Figure 10) when mutations were introduced at both G424 and G432, a result that suggested G424-AIA-G428 was a functional GXXXG motif capable of directing cis-dimerization in the absence of the two motifs centered on G432 and necessary to sustain the level of growth produced by wild type CEACAM1-4S. Although this conclusion is seemingly at odds with the 9 prediction algorithms that placed G424 outside the transmembrane domain (Table 1), we suggest that a combination of structural and functional data provides a more accurate location of the transmembrane domain, one that incorporates the GAIAG motif at the N-terminus.

Similar reasoning can be applied to the C-terminus where 5 of 12 transmembrane prediction algorithms placed Y448 outside the transmembrane domain. However, mutation of either Y445 or Y448 led to the soft agar growth enhanced phenotype, indicating first, that Y448 was functionally equivalent to Y445 and thus likely to be located within the transmembrane domain and second, that both tyrosines were required to sustain anchorage independent growth at the level produced by wild type CEACAM1-4S. However, whether Y448 was or was not in the transmembrane was moot since single Y445V or Y448V mutations produced the growth-enhanced phenotype in soft agar. Put another way, the presence of both tyrosines appeared to be necessary to suppress anchorage independent growth, a suppressive effect that apparently involved more than interactions between aromatic side chains since in both single tyrosine to valine mutants (VFLY or YFLV) the remaining tyrosine was paired with F446. Whether the two tyrosines without phenylalanine would be able maintain the wild type CEACAM1-4S phenotype is an open question that is beyond the scope of the present investigation.

While the mechanism behind the gain-of-function produced by the Y mutations is less clear, there is increasing evidence supporting the functional importance of interactions between transmembrane aromatic amino acids [41]. Although aromatic residues make up only a small percentage of the amino acids in any given protein, they are generally the most highly conserved residues. Interactions between the aromatic rings of phenylalanine, tryptophan (W) or tyrosine are thought to involve pi-stacking, a process that creates an attractive force when aromatic rings assume energetically favored stacking geometries. Accumulating evidence suggests that pi-stacking plays an important role in molecular recognition and self assembly either by contributing energy for driving self assembly or by providing directionality and orientation through stacking geometries [52], [53]. When a bacterial transmembrane database was statistically analyzed by Sal-Man et al, these investigators found that aromatic pairs (WXXW or YXXY) were significantly over-represented compared to their predicted frequency, suggesting a functional role for these sequences [52]. The stabilization of transmembrane domain self-association following substitution of tyrosine for glutamine (Q) and serine (S) in the known dimerization motif, QXXS, provided support for this idea and led these authors to conclude that stabilization of transmembrane associations by aromatic residues may be a general mechanism for generating specificity in transmembrane-transmembrane interactions. In considering which amino acid to substitute for the transmembrane tyrosine residues, phenylalanine would intuitively seem to be the logical choice. However, based on recent reports, tyrosine to phenylalanine mutations may or may not disrupt function. Stevens et al [54] reported that a YS/FA mutation of the IgM transmembrane domain had no effect on anti-Ig induced signaling as measured by the activation of tyrosine phosphorylation and did not disrupt the association between IgM and its signaling partners, Ig-alpha/Ig-beta. In contrast, a YS/VV mutation diminished both signaling and association with Ig-alpha/Ig-beta. In cases where phenylalanine substitutions fail to mimic tyrosine, the lack of a properly spaced hydroxy group may be the reason [41]. Based on these considerations, it was decided to introduce tyrosine to valine mutations.

GXXXG motifs and aromatic amino acids are common features of the transmembrane domains of many different plasma membrane proteins with GXXXG motifs most often located at the N-terminus [55], [56], [57] and Y/F/W aromatic amino acids at the C-terminus of the transmembrane domain [58], [59]. Many of the proteins also have Sternberg-Gullick dimerization motifs, a family of sequences discovered in the transmembrane domain of tyrosine kinase growth factor receptors [60]. Members of this family, which includes a subset of GXXXG/AXXXG/SXXXG dimerization motifs, are composed of 5 amino acids arranged with G, A, S, T or P in the first position (N-terminus), an A, V, L or I in the fourth position and G or A in the fifth position. Our findings demonstrate that a single amino acid substitution in the transmembrane domain of CEACAM1-4S can produce dramatic effects on cell proliferation, anchorage independent growth and *in vivo* tumorigenicity. It seems clear that the transmembrane domain and more specifically GXXXG motifs and tyrosine residues make a significant contribution to the functionality of CEACAM1-4S and by extension, to other transmembrane proteins with similar characteristics. Further studies are needed to define the downstream signaling events impacted by re-expression of CEACAM1-4S or by transmembrane domain mutations, studies that should provide valuable insights into events controlled by transmembrane domain mediated interactions. Of particular interest will be the identity of pathways inactivated/activated by tyrosine mutations that lead to the positive growth effects of tyrosine mutations and the ability of these pathways to counteract the tumor suppressor activity of CEACAM1-4L, the larger splice variant with the same transmembrane domain as the 4S isoform.
